# Silicon-coordinated nitrogen-doped graphene as a promising metal-free catalyst for N_2_O reduction by CO: a theoretical study†

**DOI:** 10.1039/c8ra03265c

**Published:** 2018-06-19

**Authors:** Anchalee Junkaew, Supawadee Namuangruk, Phornphimon Maitarad, Masahiro Ehara

**Affiliations:** National Nanotechnology Center (NANOTEC), National Science and Technology Development Agency (NSTDA) Pathum Thani 12120 Thailand supawadee@nanotec.or.th; Research Center of Nano Science and Technology, Shanghai University Shanghai 200444 P. R. China; Institute for Molecular Science Nishigo-naka 38, Myodai-ji Okazaki Aichi 444-8585 Japan ehara@ims.ac.jp

## Abstract

Metal-free catalysts for the transformation of N_2_O and CO into green products under mild conditions have long been expected. The present work proposes using silicon-coordinated nitrogen-doped graphene (SiN_4_G) as a catalyst for N_2_O reduction and CO oxidation based on periodic DFT calculations. The reaction proceeds *via* two steps, which are N_2_O reduction at the Si reaction center, producing Si–O*, which subsequently oxidizes CO to CO_2_. The N_2_O reduction occurs with an activation energy barrier of 0.34 eV, while the CO oxidation step requires an energy of 0.66 eV. The overall reaction is highly exothermic, with a reaction energy of −3.41 eV, mostly due to the N_2_ generation step. Compared to other metal-free catalysts, SiN_4_G shows the higher selectivity because it not only strongly prefers to adsorb N_2_O over CO, but the produced N_2_ and CO_2_ are easily desorbed, which prevents the poisoning of the active catalytic sites. These results demonstrate that SiN_4_G is a promising metal-free catalyst for N_2_O reduction and CO oxidation under mild conditions, as the reaction is both thermodynamically and kinetically favorable.

## Abstract 2

Mechanistic insight into the N_2_O reduction and CO oxidation on SiN_4_G is reported in this theoretical study. The high reactive and selective SiN_4_ center leads this metal-free catalyst as a promising catalyst for this reaction under mild conditions.

## Graphical abstract

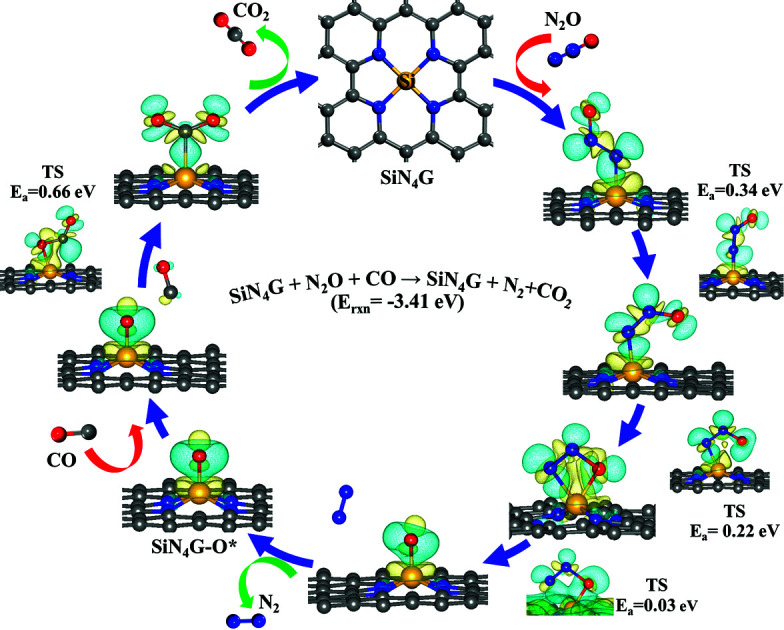

## 1. Introduction

Nitrous oxide (N_2_O) and carbon monoxide (CO) are pollutant gases which are exhausted from various combustion sources, such as vehicles and electric power plants. Because removing them requires high temperatures and large amounts of energy, catalytic materials have been extensively explored for eliminating them more efficiently. The catalytic reaction of N_2_O and CO, namely, the oxidation of CO by N_2_O or the reduction of N_2_O by CO, is one of the useful techniques for converting N_2_O and CO into less harmful products *i.e.*, N_2_ and CO_2_. In a direct N_2_O (g) + CO (g) → N_2_ (g) + CO_2_ (g) reaction, the activation barrier of 2 eV and the reaction energy of −3.6 eV were determined by a density functional theory (DFT) calculation.^1^ Although the reduction of N_2_O by CO is an exothermic reaction, it is difficult to promote in the gas phase due to the large activation energy (*E*_a_) required.^2,3^

In the literature, various metals and metal ions such as Pt^+^, Ir^+^, Os^+^, Fe^+^, Ca^+^, Ge^+^, Sr^+^, Ba^+^, Eu^+^, and Yb^+^ have been explored for use as catalysts in the elimination of CO and N_2_O at low temperatures.^4,5^ Also, the reduction of N_2_O by CO have been investigated on metal cluster catalysts such as Pt_*n*_^−^ (*n* = 3–4)^2,6^ neutral Rh_*n*_ (*n* = 10–28),^7^ neutral Pt_*n*_ (*n* = 4–12),^8^ Pt_*n*_^+^ (*n* = 6–8),^9^ Cu anions,^10^ Cu_*n*_ (*n* = 4–15),^1^ Ag_7_Au_6_ clusters,^11^*etc.* Although metal-based catalysts perform well when eliminating CO and N_2_O at low temperatures, their mass-scale uses are economically limited due to their cost. Various catalytic materials, such as metals on supports, metal complexes, metal oxides and metal-free catalysts have been sought in order to reduce costs while retaining good catalytic performance.^12–17^

Recently, nitrogen-doped graphene (NG) became a promising metal-free catalyst; it had been proposed for various catalytic applications, such as electrochemical, oxidation and other reactions.^18–21^ A four pyridinic-N defect with a di-vacancy in graphene (*i.e.* 4N + DV) is one of the pyridinic forms that can be drawn from past experiments.^22^ The pyridinic-N defect provides a greater chemical reactivity than the graphitic-N and it can trap a single metal atom, such as Mg, Al, Ca, Ti, Cr, Mn, and Fe.^22^ This 4N + DV defective graphene (N_4_G) has a porphyrin-like core. Varying doping species at the porphyrin-like core can fine-tune its properties for desired applications. For instance, FeN_4_G, CoN_4_G, NiN_4_G, and MnN_4_G have been applied for the oxygen reduction reaction (ORR) applications.^23–26^ Later, the possible use of FeN_4_G as a catalyst for NO reduction *via* the (NO)_2_ adsorption mechanism was suggested by the periodic DFT method.^27^ Very recently, water dissociation on MN_4_G (M = Mg, Ba, Ti) was theoretically studied by Liu *et al.*^28^ They showed that Mg, Ba, Ti are energetically stable on the surface and TiN_4_G displayed promising catalytic properties for H_2_O dissociation.

For applications involving N_2_O and CO removal, p-block element doped into carbon and other support materials have been used as catalysts.^13,15–17,29^ Since Si is the second most abundant element on Earth,^30^ it is attractive to corporate with other elements for applying in various applications. From previous experimental work, Si coordinated with four nitrogen atoms has been proposed as the active site in Si-porphyrin and Si-corroles.^31,32^ Si-porphyrin was successfully synthesized^32^ and it has been further applied in a photochemical oxygenation of alkenes,^30^ water oxidation to hydrogen peroxide,^33^ and dye-sensitized solar cell applications.^34^ For Si-corroles, Si-porphyrine derivatives, provides good luminescence properties and they have been applied as a sensor for F^−^ detection.^31^ Interesting work has been reported recently by Tang *et al.*; they found that Si-coordinated nitrogen-doped graphene (SiN_4_G), which is a metal-free catalyst, had catalytic properties that promoted CO oxidation by O_2_.^35^ These reports noted that Si atoms doped into 2D materials are a reactive dopant for CO and N_2_O gases. However, it is unclear why a Si dopant, which is a non-metal atom, was specifically reactive to those gases.

Moreover, to the best of our knowledge, Si doped into N_4_G for the reduction of N_2_O by CO has not been studied so far. Thus, we are motivated to ask: How feasible is this metal-free catalyst SiN_4_G for the reduction of N_2_O by CO? Is it possible to use this catalyst in a low temperature range? In this work, these questions are answered systemically using a plane-wave-based DFT investigation. The gas adsorption and detailed reaction mechanisms were examined. Moreover, the performance of SiN_4_G is compared with other catalysts from the literature in order to determine the feasibility of using this catalyst for the reduction of N_2_O by CO. The results will be valuable for developing low cost catalysts for pollutant gas abatement applications.

## 2. Method

**Fig. 1:**
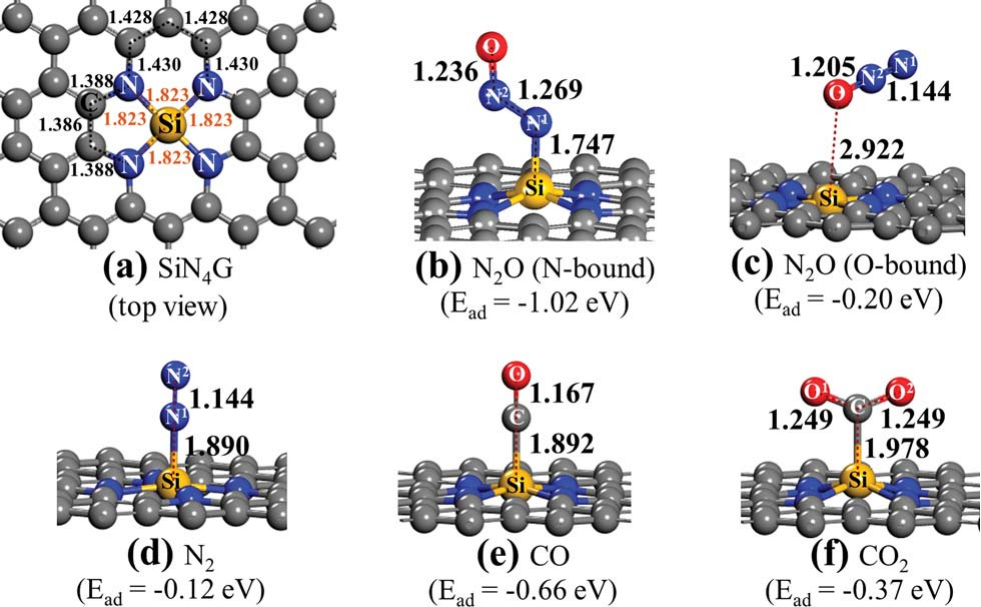
Structures of (a) SiN_4_G, (b) SiN_4_G/N_2_O (N-bound), (c) SiN_4_G/N_2_O (O-bound), (d) SiN_4_G/N_2_, (e) SiN_4_G/CO and (f) SiN_4_G/CO_2_. Selected bond lengths (Å) and *E*_ad_ values of adsorbed gas molecules on the catalyst are given.

The gas adsorption and the reaction mechanism of reduction of N_2_O by CO over SiN_4_G were examined by using plane-wave-based DFT calculations implemented in the Vienna *ab initio* simulation package (VASP version 5.4.1).^36,37^ The projector-augmented wave (PAW)^38^ with a generalized gradient approximation (GGA) refined by Perdew, Burke and Ernzerhof (PBE)^39^ was used in this work. Grimme's DFT-D3 was applied for the dispersion contribution.^40^ The 1 × 10^−5^ eV per cell and 5 × 10^−3^ eV Å^−1^ were set as the energy- and force-convergence parameters, respectively. An energy cutoff of 400 eV and a spin-unrestricted calculation were applied in all cases. The SiN_4_G slab was constructed by placing one Si at the N_4_ center of a (5 × 5) slab of graphene with *a* = 12.4 Å and *b* = 12.2 Å. The slab was separated by 15 Å of vacuum. This size of the simulated cell is large enough to prevent interfering of adsorption interaction by its replicas. All atoms in the cell were relaxed during the calculations. After the optimized SiN_4_G was obtained, the simulated cell was fixed during gas adsorption and reaction mechanism calculations. A top view of a center site of SiN_4_G is illustrated in Fig. 1a. The isolated gas molecule was placed in the 15 Å × 15 Å × 15 Å box. The Monkhorst–Pack grids of 5 × 5 × 1 and 1 × 1 × 1 were applied for slab and isolated gas structures, respectively.

The transition state (TS) of each elementary step was calculated from the climbing-image nudged elastic band (CI-NEB)^41,42^ and the dimer method.^43,44^ The criterion of the force convergence threshold was 0.025 eV Å^−1^. Each TS structure was confirmed by its single imaginary frequency. The projected density of state (PDOS), electron density difference and Bader charge analyses^45,46^ were elucidated in this work.

## 3. Results and discussion

### 3.1 Gas adsorption on SiN_4_G

First, the adsorption properties of reactants and products over SiN_4_G are discussed. The adsorption energy (*E*_ad_) is calculated by

1 *E*_ad_ = *E*^complex^_adsorbate–substrate_ − *E*^isolated^_substrate_ − *E*^isolated^_adsorbate_

where *E*^complex^_adsorbate–substrate_, *E*^isolated^_substrate_and *E*^isolated^_adsorbate_ are the total energies of the gas adsorbed the SiN_4_G complex, bare SiN_4_G surface and isolated gas, respectively. A negative *E*_ad_ value means stronger adsorption and greater stability of the gas-adsorbed surface complex. The calculated *E*_ad_ values and relevant interatomic distances of the adsorbed N_2_O, N_2_, CO and CO_2_ on SiN_4_G are given in Fig. 1.

Fig. 1a shows the top view structure of the optimized bare SiN_4_G sheet. The bond distances around the core site of SiN_4_G are given. The Si atom is surrounded by two five-membered rings and two six-membered rings. The four Si–N bond lengths are equivalent at 1.823 Å. According the calculated *E*_ad_ values expressed in Fig. 1, the order of the binding strength of the four gas species on the catalyst is N_2_O > CO > CO_2_ > N_2_. The linear N_2_O gas strongly adsorbs on the Si atom with the N-bound mode resulting in a bent N–N–O conformation and the calculated *E*_ad_ is −1.02 eV (see Fig. 1b). Both N–N and N–O bonds are lengthened from 1.146 Å and 1.200 Å (free N_2_O gas) to 1.269 Å and 1.236 Å, respectively. The formed coordination bond between N and Si is 1.747 Å. In Fig. 1c, the O-bound mode is less favorable for N_2_O adsorption. In the N_2_ case, it shows the least binding stability with *E*_ad_ ∼ −0.12 eV as shown in Fig. 1d. It is worth to note that the attached N_2_ at Si site in Fig. 1d is slightly less stable than the detached N_2_ (*E*_ad_ = −0.17 eV) presented in Fig. S1a of ESI.†

For CO and CO_2_, their carbon atoms attach to the Si atom and their binding energies are −0.66 and −0.37 eV, respectively. By comparing their *E*_ad_ values, we can see that the CO adsorption in Fig. 1e is stronger than the CO_2_ adsorption in Fig. 1f. The end-on configuration of adsorbed CO over SiN_4_G agrees well with literature.^35^ The C–O bonds are activated in both CO and CO_2_ compared with the free molecules. The *E*_ad_ values from the prior work are −0.55 eV and −0.26 eV for CO and CO_2_ adsorption, respectively. Our calculated *E*_ad_ values are slightly stronger than those of the previous calculations because the dispersion correction is included in this work. As a result, the Si site moves along the out-of-plane direction when it chemically adsorbs molecules. On the other hand, the SiN_4_G sheet is not changed when it binds with gas through physisorption interaction (see Fig. 1c).

A different order of the adsorption energies was observed in the adsorption of these gasses on Si-doped graphene (SiG) studied by Gholizadeh and Yu.^15^ Their calculated *E*_ad_ values are small and are in the range of −0.2 eV to −0.15 eV and the adsorption strength follows the order of SiG/CO > SiG/CO_2_ > SiG/N_2_O > SiG/N_2_. Therefore, the coordinated Si with nitrogen at the active center site in the present SiN_4_G significantly enhances the adsorption ability and changes the order of these gas adsorptions. Due to the distinctly stronger interaction of N_2_O on SiN_4_G when compared to CO on SiN_4_G, the N_2_O reduction would occur first, rather than the CO oxidation. Therefore, the present results of these adsorption energies also support the claim that the reaction cycle can proceed as the two sequential steps: (1) N_2_O → N_2_ + O* and (2) O* + CO → CO_2_, respectively. The CO_2_ and N_2_ products can be easily desorbed due to their low binding strength. This advantage can prevent catalyst poisoning by the products and allows the active site to get ready to react with the reactant in the next cycle.

**Fig. 2:**
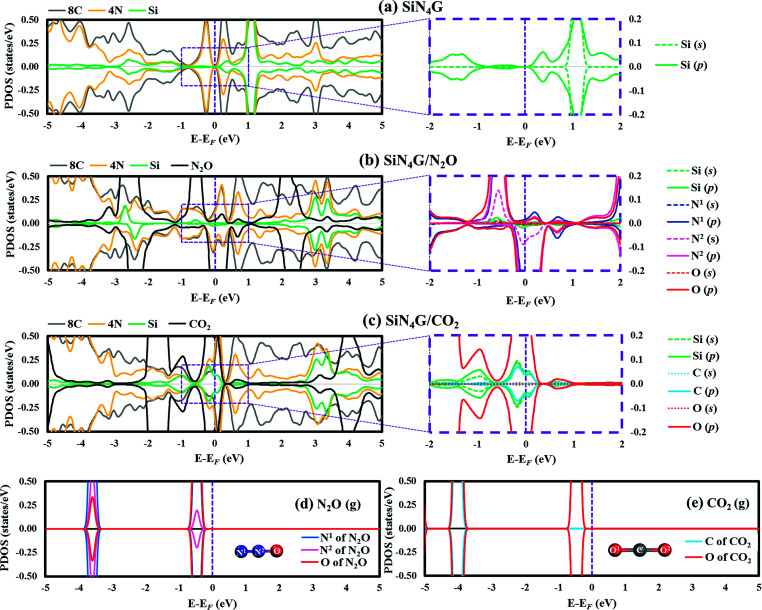
PDOS plots of selected atoms in (a) bare SiN_4_G, (b) SiN_4_G/N_2_O, (c) SiN_4_G/CO_2_, (d) isolated N_2_O gas and (e) isolated CO_2_ gas. In (a) to (c), the PDOS peaks of Si, 4N, 8C and gas molecules are presented in the left panels, and the s- and p-states of selected Si and atoms of gas molecules are decomposed in the right panels. Positive and negative amplitudes of PDOS indicate the spin-up and spin-down states, respectively.

The projected density of states (PDOSs) of bare SiN_4_G, and the adsorbed N_2_O and CO_2_ on the SiN_4_G structures, were analyzed and compared in Fig. 2. The Fermi level (*E*_F_) is shown by the vertical dashed line at 0 eV. Positive and negative amplitudes of PDOS correspond to the spin-up and spin-down states, respectively. Fig. 2a depicts the PDOS plot of SiN_4_G. The hybridization of the valence states and bonding can be seen in overlapping peaks. In addition, PDOS analysis of the pyridinic-N embedded in the graphene (N_4_G) is depicted in Fig. S2 in ESI.† The PDOS of N_4_G agrees well with those reported in other literatures.^47,48^ The PDOS peaks of the Si, the four pyridinic-N atoms (4N) and the eight neighbouring carbon atoms around the N atoms (8C) of the bare SiN_4_G are depicted in Fig. 2a. The right panel of Fig. 2a shows the PDOS of the decomposed s- and p-states of Si around *E*_F_, which are relevant for the reaction. SiN_4_G has symmetrical spin-up and spin-down peaks. The overlapped valence and conduction bands of Si and N atoms, with their adjacent carbons, can be seen in Fig. 2a. In SiN_4_G, the p-states of Si located close to the *E*_F_ level can be found in the right panel of Fig. 2a. Furthermore, the PDOS plots of N_2_O and CO_2_ adsorbed on SiN_4_G are also shown in Fig. 2b and c. The chemical bonds between Si and the adsorbed molecules can be observed *via* the hybridization of their PDOS peaks. To see the variation of PDOS of the adsorbed gas species, PDOS plots of isolated N_2_O and CO_2_ molecules are also provided in Fig. 2d and e, respectively. For SiN_4_G/N_2_O, we found the coupling between the p-states of Si and the valence p-states the N^1^ of N_2_O (g) near *E*_F_ results in hybridization peaks at *E*_F_ (right panel of Fig. 2b). This signifies the bonding between N_2_O and SiN_4_G. SiN_4_G donates an electron to N_2_O resulting in asymmetrical spin-up and spin-down peaks around *E*_F_ (see Fig. 2b).

In contrast to SiN_4_G/N_2_O, SiN_4_G/CO_2_ shows a symmetrical nature. The broad hybridized peaks can be observed in Fig. 2c. By comparing them with the PDOS of bare SiN_4_G, the reduction of the occupied valence states can be observed. The PDOS peaks of Si and the gas are decomposed into their s- and p-states and are illustrated in the right panel of Fig. 2c. The overlapping states of Si and CO_2_ can be seen at *E*_F_. The PDOS of only one oxygen atom of CO_2_ is presented in Fig. 2c, since the two oxygen atoms in CO_2_ are identical to each other. According to Fig. 2c, the overlapped peaks around *E*_F_ are the hybridization of the states near *E*_F_ of Si in bare SiN_4_G (see Fig. 2a) and the states of isolated CO_2_ (Fig. 2e).

As a result, the hybridization between the states of Si and the states of N_2_O and CO_2_ results in chemical bonds between Si and those gas molecules. In addition, the Bader charge results were also calculated in this work. The changes in the valence electrons (Δe^−^) of the selected atoms are given in Table S1 in ESI.† Our Bader charge results demonstrate that N_2_O and CO_2_ gain more electrons from SiN_4_G, approximately 1.03|e| and 1.06|e|, respectively. The electronegativity of O, which is greater than those of N, C and Si, also influences the direction of electron transfer in gas adsorbed SiN_4_G. Therefore, both the PDOS and Bader charge results support the claim that electrons are transferred from SiN_4_G to those adsorbed gas molecules.

### 3.2 Mechanisms

The detailed mechanism of the N_2_O reduction by CO is discussed in this section. The overall reaction can be described by eqn (2). There are two possible mechanisms for this reaction, which are the concerted- and stepwise-mechanisms. The concerted mechanism presents the reaction that N_2_O and CO interact at once to produce N_2_ and CO_2_. In the stepwise mechanism, the N_2_O reduction over SiN_4_G occurs first followed by CO oxidation over SiN_4_G-O* as represented by eqn (3) and (4), respectively.

2 N_2_O (g) + CO (g) → N_2_ (g) + CO_2_ (g)

3 N_2_O (g) → *N_2_O → N_2_ (g) + O*

4 O* + CO (g) → *CO_2_ → CO_2_ (g)

where an asterisk (*) expresses an adsorbed intermediate on a catalyst. The energy profiles of the proposed mechanisms are compared to find the most favorable pathway. The reaction energy and the activation energy (*E*_a_) of the rate-determining step are used to determine the feasibility of using this catalyst for the N_2_O and CO elimination.

#### 3.2.1 Concerted mechanism

**Fig. 3:**
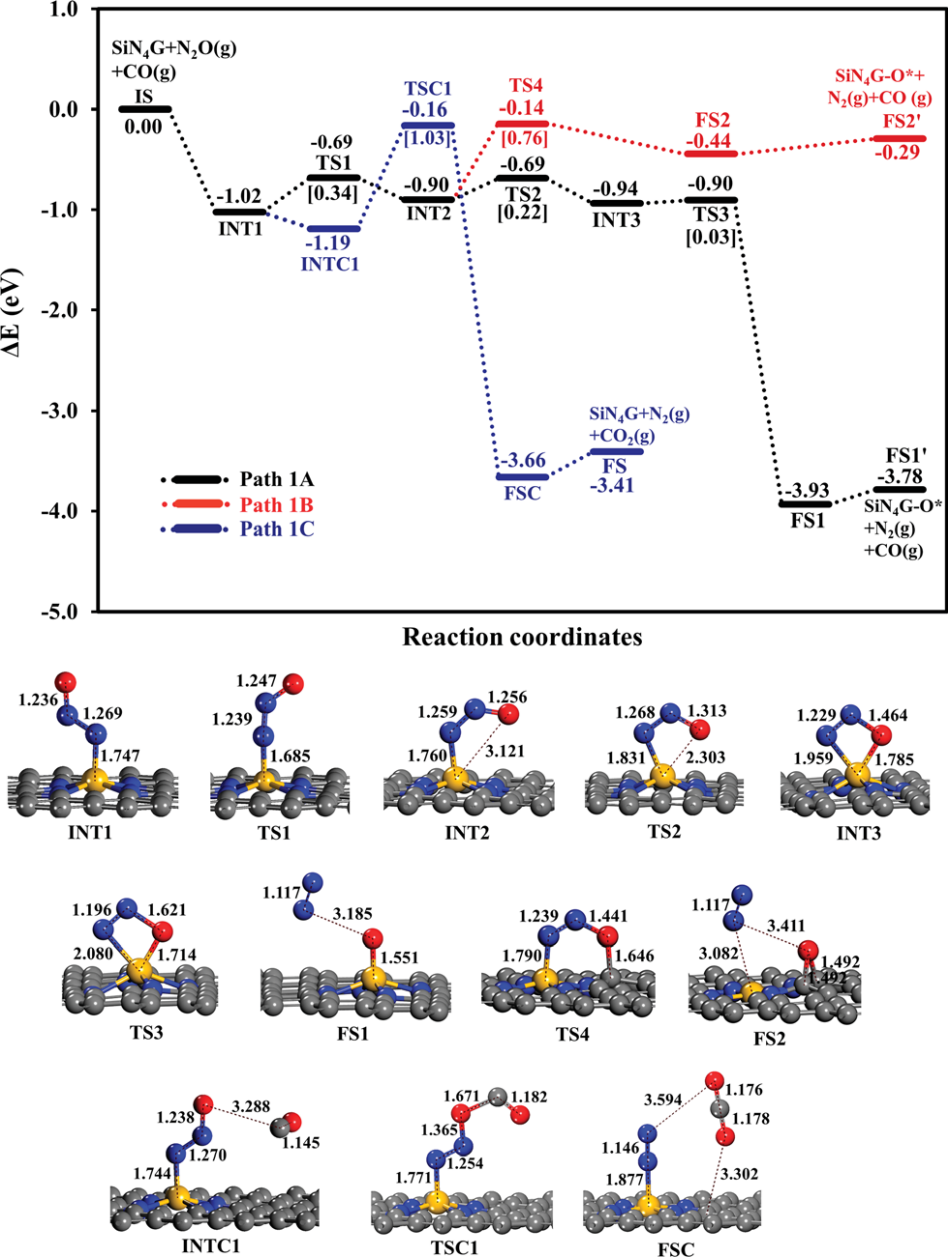
Energy profiles of Path 1A (black line), Path 1B (red line) and Path 1C (blue line) with the corresponding structures.

The N_2_O reduction starts from the N_2_O adsorption and the N_2_O dissociation. As discussed above, N_2_O interacts with the Si active site by the N-bound mode more strongly than the O-bound mode. Thus, our proposed pathways focus on the N-bound mode of N_2_O adsorbed SiN_4_G. After N_2_O adsorbs at the Si active site forming INT1, the CO molecule is not able to adsorb at the same Si active site with the pre-adsorbed N_2_O as Langmuir–Hinshellwood (LH) manner, but rather weakly interacts with the pre-adsorbed N_2_O as Eley–Rideal (ER) one with some possible configurations of very low adsorption energies ranging from −0.10 to −0.17 eV (see INTC1 in Fig. 3 and S1f in ESI†). These weak adsorption energies of INTC1 configurations imply that the possibility of CO involved in the reaction in the concerted mechanism is rare. Nonetheless, the energetics of concerted pathway *via* the ER mechanism has been examined. The energy profile is presented by the blue pathway in Fig. 3. The configurations in Fig. 3 present that CO interacts with the pre-adsorbed N_2_O and abstracts O atom from N_2_O, and then CO_2_ desorbs at the FSC to FS step. Although this process is exothermic, the high *E*_a_ of 1.03 eV which is required to surmount the TSC1 state and the low probability of forming INTC1 prohibit this concerted pathway.

#### 3.2.2 Stepwise mechanism

##### N_2_O reduction

Two possible pathways of the N_2_O dissociation are compared for the stepwise mechanism. The first pathway (Path 1A) represents the conversion of N_2_O to N_2_ and leaves its O attached on the Si-site. In another possible pathway (Path 1B), the leaving O atom of N_2_O is bound at the C–C bridge site of the SiN_4_G catalyst.

The relative energy profile of Path 1A with structures and selected bond distances are shown as the black profile in Fig. 3. The total energies of the bare SiN_4_G, isolated N_2_O and isolated CO are used as a reference to calculate the relative energy (Δ*E*). The *E*_a_ value of each transition state is given in the bracket. This pathway starts from the N_2_O adsorption intermediates of INT1 and INT2 with an *E*_ad_ of approximately −1 eV. The transformation of INT1 to INT2 needs energy barrier of 0.34 eV at the TS1 state. The N_2_O molecule is bent and distorted on SiN_4_G, in particular for INT2 with the terminal O atom directed to the Si center. For the INT2 → TS2 → INT3 steps, a small *E*_a_ of 0.22 eV is required to enable the bonding between Si and O atoms; at TS2, the S–O distance decreases to 2.303 Å to form a bond and is 1.785 Å at INT3. The N–O bond is then broken simultaneously with a negligible *E*_a_ of 0.03 eV at TS3. Finally, N_2_ is produced and the O atom remains at the Si site at the final state (FS1) of this elementary step. The Si–O bond length of FS1 is 1.551 Å. The energy difference between the FS1 and FS1′ describes that the N_2_ is easily desorbed from SiN_4_G-O* with very little energy, approximately 0.15 eV. In addition, Path 1A is a highly exothermic reaction with a reaction energy of −3.78 eV.

Another possible route (Path 1B) is examined, and its energy profile with structures is depicted by the red profile in Fig. 3. This pathway proceeds through the INT2 → TS4 → FS2 steps. An *E*_a_ of 0.76 eV is required to surmount the energy barrier at TS4. Both N–O and N–Si bond lengths are elongated at TS4 in order to release N_2_ as a product. For the TS4 to FS2 step, the dissociated O atom binds on atop C site at TS4. Then N_2_ is simultaneously desorbed from Si and the dissociated O atom moves simultaneously towards the C–C bridge site, which is more energetically favorable than the top C site, at the final state (FS2). According to the relative energies, the SiN_4_G-O* structure of FS2, in which the O atom is attached to the C–C bridge site, is less stable than that of FS1, where the O atom binds with the Si site. Unlike Path 1A, this pathway is an endothermic reaction due to the less stable FS2.

In summary, Path 1A is more kinetically and thermodynamically favorable than Path 1B. The activation barrier of N_2_O dissociation on SiN_4_G for the N_2_O reduction is less than that on SiG (*E*_a_ ∼ 0.5 eV).^15^ Thus, the surrounding N atoms at the Si center not only improve the adsorption ability of N_2_O adsorption but also enhance the activity of catalyst in the N_2_O dissociation process.

##### CO oxidation

**Fig. 4:**
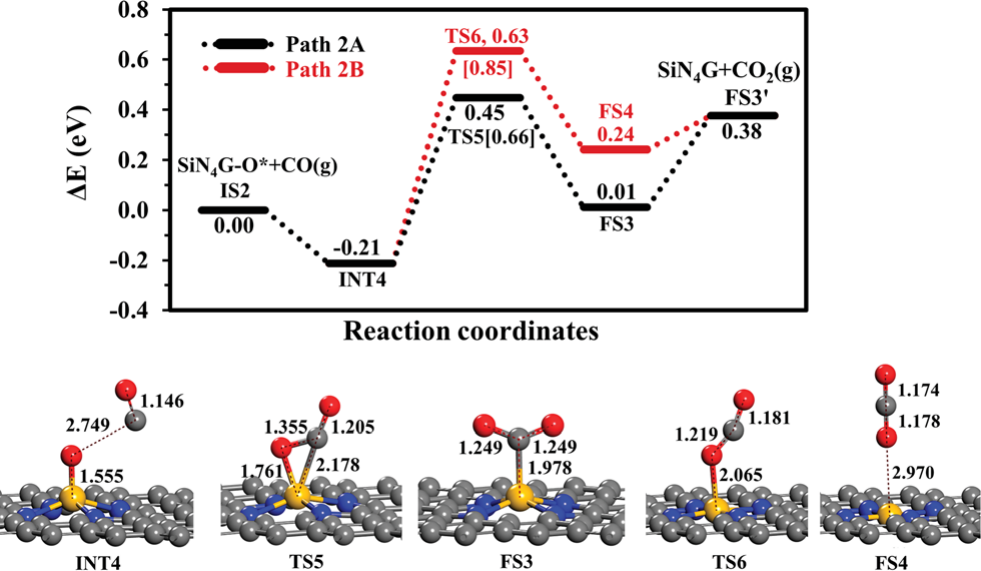
Energy profiles of Path 2A and 2B with the corresponding structures.

The second step is the CO oxidation, in which CO is oxidized by the pre-adsorbed oxygen from the previous step to produce CO_2_, as outlined in eqn (4). The energy profile of this step and the structures are displayed in Fig. 4. Two competitive pathways, Path 2A and Path 2B, are considered. Both of these pathways start from INT4 where the CO molecule weakly interacts with SiN_4_G-O*. In Path 2A, CO interacts with O* and then forms the CO_2_ product attached on the Si site with C atom. The energy barrier of the INT4 → TS5 → FS3 step is 0.66 eV and this step is slightly endothermic by +0.01 eV relative to IS2. Then CO_2_ is desorbed directly from FS3 to FS3′. This step requires energy approximately 0.37 eV. The PES and corresponding configurations of the direct desorption CO_2_ desorption process, the FS3 to FS3′ step, are shown in Fig. S5 in ESI.†

In Path 2B, CO interacts with O* and release CO_2_ from Si site simultaneously (see FS4 in Fig. 4). The energy barrier to surmount TS6 is 0.85 eV. For this state, CO_2_ needs 0.14 eV to desorb from the catalyst in FS3′. Similar to Path 2A, the CO_2_ desorption energy is less than the energy of breaking the O–CO bond. This step is also endothermic. Consequently, Path 2A is more favorable than Path 2B in view of energetics. The present *E*_a_ value of Path 2A is lower by approximately 0.1 eV than the reported one by Tang *et al.* in the study of CO oxidation by O_2_.^35^ In summary, the CO oxidation step is less thermodynamically and kinetically preferable than the N_2_O reduction step, however, this reaction is feasible at low temperatures, which is supported by the calculated activation energy barriers and reaction energy. Finally, SiN_4_G is completely regenerated and ready for the next N_2_O reduction.

**Fig. 5:**
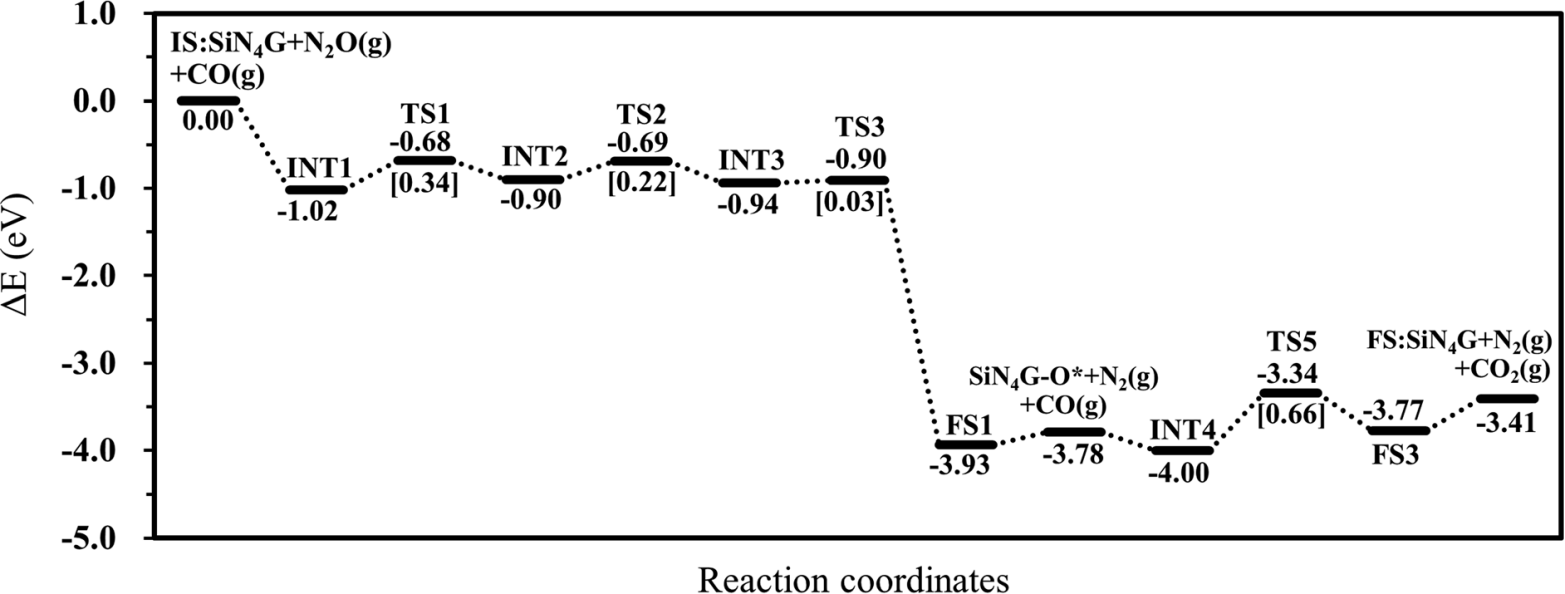
Energy profile of the most favorable pathway of the N_2_O reduction by CO on SiN_4_G.

To conclude all the results in this work, the N_2_O reduction by CO prefers the stepwise mechanism than the concerted mechanism. The energy profile of the most favorable stepwise pathway of the N_2_O reduction by CO on SiN_4_G is presented in Fig. 5. A summation of the energies of bare SiN_4_G, N_2_O and CO is used as the reference energy. The calculated reaction energy of N_2_O + CO → N_2_ + CO_2_ in this work is −3.41 eV. This value is comparable to −3.5 eV reported by theoretical calculations in Si- and Fe-doped graphene^15,49^ and −3.8 eV of the direct reaction of CO with N_2_O from experiment.^50^ The N_2_O reduction and CO oxidation prefer Path 1A with an *E*_a_ of 0.34 eV and Path 2A with an *E*_a_ of 0.66 eV. Thus, the rate-determining step of the overall reaction is the CO oxidation. It is worth mentioning that N_2_O is adsorbed more strongly than CO_2_, as seen from their adsorption energies. The small adsorption energies of the N_2_ and CO_2_ products in this system indicate that the products easily desorb from the active site, which prevents them from poisoning the catalyst. Hence, the active SiN_4_G site can be recovered after the reaction is completed and the next reaction cycle can continue further. Overall, the SiN_4_G catalyst demonstrates a promising performance for the reduction of N_2_O by CO.

To form a better understanding of the charge property of the systems in the reaction, electron density differences of the intermediates and transition states along the most favorable pathway are also investigated. The results are given in Fig. S3 and S4 in ESI.† For bare SiN_4_G, the electron density difference is referenced with the isolated Si atom and N_4_G surface, see Fig. S3 in ESI.† The light blue and yellow regions represent the electron density increment and reduction, respectively. The electron density of Si is depleted when Si is embedded at the porphyrin-like core and is delocalized over N atoms and the Si–N bonds around the di-vacancy site. This result agrees well with the Bader charge analysis in Table S1 in ESI;† the partial charge of Si is about +2.66|*e*| and the coordinating N atoms show an average negative charge about −1.5|*e*|. Therefore, these four N atoms strongly coordinate with the embedded Si atom as confirmed by the adsorption energy of Si on N_4_G of −7.07 eV. This value is comparable with −7.25 eV, reported by Tang *et al.*^35^ This strong adsorption stabilizes the catalyst and prevent the leakage of the embedded Si atoms from the catalyst, resulting in increasing the durable operation. When all adsorbed species attached on SiN_4_G, electrons are obviously transferred from Si and accumulated between Si and the attached atom (see Fig. S4 in ESI†). This electron transfer supports the PDOS result.

### 3.3 Comparison with other two dimensional catalysts

Herein, the expected catalytic activity of SiN_4_G for the reduction of N_2_O by CO is considered in comparison with the Si-based graphenes or nanotubes and other metal-based catalysts from previous reports. The activation energies of the rate-limiting steps of the N_2_O reduction and the CO oxidation of some catalysts are compared in Table 1. The effect of the coordinating N atoms in the SiN_4_G on the catalytic activity of the catalyst can be described by comparing SiN_4_G with SiG.^15^ SiN_4_G has a lower *E*_a_ in the N_2_O reduction step but a higher *E*_a_ in the CO oxidation step. For the entire N_2_O and CO_2_ reaction, the *E*_a_ of the rate determining step is 0.5 eV in SiG, which is slightly lower than that of SiN_4_G. However, as mentioned in Section 3.1, the CO adsorption on SiG is slightly stronger than that of N_2_O, which indicates that the selectivity of SiG towards the N_2_O reduction is poorer than SiN_4_G if CO exists in the system. Thus, the presence of N atoms around the Si does not only enhance the adsorption with the reactants, but it also makes the catalyst become more selective. In SeG, the *E*_a_ of the N_2_O reduction is approximately 1.8 eV which is much higher than the Si doping case.^15^ Note that for SiN_4_G, the adsorption CO_2_ product is not comparative to the adsorption of the CO and N_2_O reactants. This characteristic is different from the SeG and SiG systems, in which reactants and products show similar adsorption energies. This means that the CO_2_ product in the case of SeG and SiG may poison the active sites, while in the case of SiN_4_G the CO_2_ is readily desorbed to get the active site ready for the next reaction cycle.

**Table 1:** Comparison of the rate-limiting steps and corresponding activation energy barriers (*E*_a_) in eV for the N_2_O and CO reactions on catalysts calculated by DFT method at 0 K

Catalyst	*E*_a_ (eV)		*E*_ad_ (eV)		
	N_2_O → N_2_ + O*	CO + O* → CO_2_	N_2_O	CO	CO_2_
SiN_4_G (this work)	0.34	0.66	−0.97	−0.66	−0.37
SiN_4_G^35^		0.72		−0.55	−0.26
Mn-N_4_ carbon nanotube (MnN_4_CNT)^51^		1.49		−2.20	
Si-doped graphene (SiG)^15^	0.5	0.3	−0.18	−0.19	−0.18
Se-doped graphene (SeG)^15^	1.8	0.7	−0.22	−0.18	−0.21
Si-doped boron nitride nanotubes (Si_B_-BNNTs)^13^	BL^a^	0.08	N/A	−0.19	
Si-doped boron nitride nanotubes (Si_N_-BNNTs)^13^	BL^a^	0.42	N/A	−0.16	
Silicon carbide nanotubes ((6,0)-SiCNT)^17^	0.71	1.01	−0.64	−0.38	
Silicon carbide nanosheets (SiCNS)^17^	1.12	0.98	−0.59	−0.18	−0.14
Pd-doped graphene (PdG)^52^		0.26		−1.04	−0.21
Al-doped graphene (AlG)^16^	0.24	0.06	−0.81	−0.62	−0.52
Ti-doped graphene (TiG)^16^	BL^a^	0.16	N/A	−1.03	−0.32
Fe-doped graphene (FeG)^49^	0.4	0.2	−0.4	−1.5	−0.4
Pd-doped boron nitride (PdBN)^53^		0.23		−1.07	−0.06
Ag-doped boron nitride (AgBN)^54^		0.17		−1.04	−0.36
Co-doped boron nitride (AgBN)^55^		0.16		−1.04	−0.33
Ag_6_Au_7_ cluster^11^	1.1	0.5	−0.2	−0.5	−0.1
Cu_7_ cluster^1^	BL^a^	0.9	N/A		
Cu_12_ cluster^1^	BL^a^	0.8	N/A		

a BL denotes a barrierless process.

Moreover, the CO oxidation on Si-doped BN nanotubes (Si-BNNTs) and Ti-doped graphene (TiG), and the N_2_O reduction by CO on Al-doped graphene (AlG) and Fe-doped graphene (FeG) are facile based on their activation energies. In the Fe-doped graphene (FeG), the rate-limiting steps require 0.4 eV and 0.8 eV for the stepwise and concerted mechanisms, respectively. However, the N_2_O adsorption on FeG (*E*_ad_ ∼ −0.4 eV) is less favorable than the CO adsorption on FeG (*E*_ad_ ∼ −1.5 eV).^49^ Thus, FeG is not selective to the present reaction. As presented in Table 1, many metal-free catalysts were proposed to show better catalytic performance than pure metal cluster catalysts like Cu- and Ag_6_Au_7_-clusters.^1,11^

In conclusion, the results in this work indicate that SiN_4_G is a promising catalyst because of the following reasons: (1) the durability of the catalyst indicated by the substantial adsorption strength of Si on N_4_G (−7.07 eV), (2) the high reactivity and selectivity for converting N_2_O and CO to less harmful products, N_2_ and CO_2_, at low temperature, indicated by low activation barriers and large reaction energy, (3) the small adsorption energy of the CO_2_ product prevents catalyst poisoning, which is a problem with most of the conventional metal catalysts, and (4) a low cost for large-scale reactions in industry.

## 4. Conclusion

This work investigated the charge, density of state, energetic and structural properties for the reduction of N_2_O reduction by CO on the Si-coordinated nitrogen-doped graphene catalyst, SiN_4_G, by employing plane-wave-based DFT calculations. The mechanistic insight of the N_2_O and CO reaction has been systematically examined. The calculation results show that the four coordinating N atoms of SiN_4_G increase the reactivity of the Si active site and stabilize the adsorption state by charge transfer. The Si active site is specifically reactive to N_2_O due to the strong overlap between the Si active site and the N atom of the adsorbed molecule. The reaction mechanism for the reduction of N_2_O by CO occurs through two consecutive steps: N_2_O reduction followed CO oxidation. The overall reaction is thermodynamically and kinetically preferable due to its low activation barriers and exothermic characteristic. The N_2_O reduction occurs easily, with a small energy barrier of 0.34 eV, while the CO oxidation is the rate determining step of the entire reaction, with an *E*_a_ of 0.66 eV and slight endothermicity. However, CO_2_ can be released more easily than it can be dissociated back to CO. The SiN_4_G is regenerated completely after CO oxidation. Then, a new reaction cycle occurs simultaneously due to the stronger interaction between SiN_4_G and N_2_O when compared with the CO product. This catalyst also shows strong reaction activity when compared to proposed catalysts from the literature. In summary, this metal-free catalyst is one of many candidates for eliminating CO and N_2_O at low temperature, and it is reusable for multiple reaction cycles. The mechanistic insight from this work provides a useful guidance in designing a low-cost metal-free catalyst with great catalytic performance.

## Conflicts of interest

There are no conflicts to declare.

## Supplementary Material

RA-008-C8RA03265C-s001
