# Supplementary material for: Silicon-coordinated nitrogen-doped graphene as a promising metal-free catalyst for N2O reduction by CO: a theoretical study
Source: RSC Adv. 2018 Jun 19;8(40):22322–30. doi: 10.1039/c8ra03265c (PMC9081867; doi:10.1039/c8ra03265c)
Supplement: RA-008-C8RA03265C-s001 [file RA-008-C8RA03265C-s001.pdf]

Electronic Supplementary Material (ESI) for RSC Advances.
This journal is © The Royal Society of Chemistry 2018

Electronic Supplementary Information

Silicon coordinated nitrogen-doped graphene as a promising metal free catalyst for N_2O reduction by CO: A theoretical study

Anchalee Junkaew,[a] Supawadee Namuangruk,*[a] Phornphimon Maitarad [b] and Masahiro Ehara *[c]

1. Gas adsorption

The additional adsorption results are provided in this part. For Fig. S1(a) to S1(f), the E_{ad} value of each structure was calculated by using the summation of the total energies of SiN_4G and isolated adsorbate as the reference energy. In Fig. S1(f), E_{ad1} was calculated from the summation of the total energies of SiN_4G, isolated N_2O gas and isolated CO gas as the reference. The E_{ad2} value was obtained from the total energies of pre-adsorbed N_2O on SiN_4G and isolated CO as the reference.

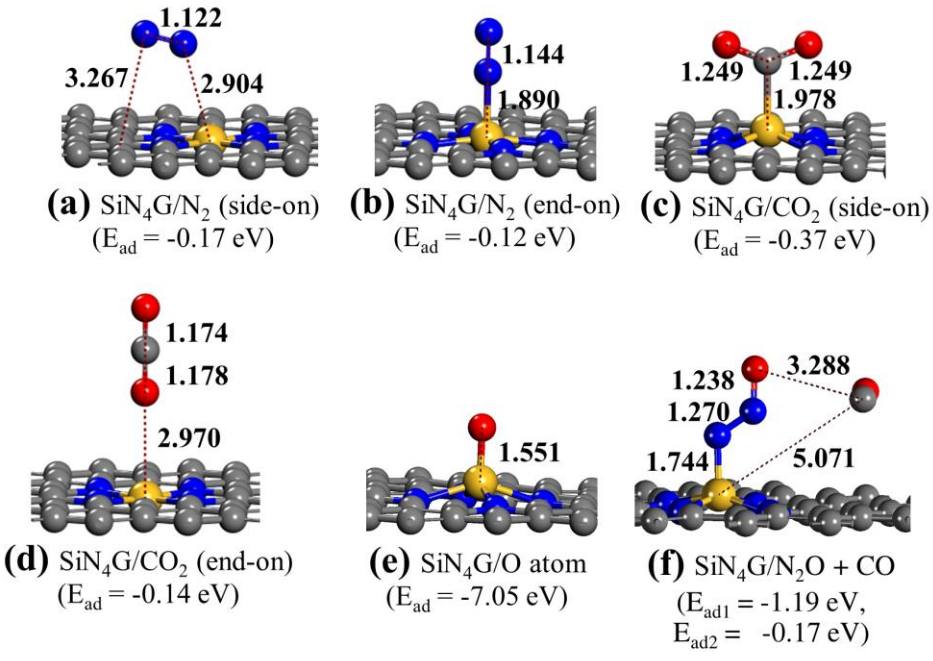

Figure S1. Adsorption structures of molecules and atom on SiN_4G and SiN_4G-O*

2. Electronic charge analysis

The PDOS analysis demonstrated the distribution and contribution of the electronic states at particular energy levels. The charge of each atom in the system was determined by Bader charge analysis. The electron density difference describes the change in electron densities when a molecule or an atom is attached to a substrate compared to their separated phases. These three parameters can be used to understand the nature of the electronic properties and interactions in the systems.

In **Fig. S2**, the electron density difference of each system is referenced with the bare SiN_4G catalyst and the adsorbed species. For INT1 in **Fig. S4**, the electron transferred from the four coordinating N atoms through Si atom to the adsorbed N_2O after the adsorption of N_2O. This aspect corresponds well with the reduction of the Bader charge and the electron density of the four N and Si atoms, shown in **Table S1**.

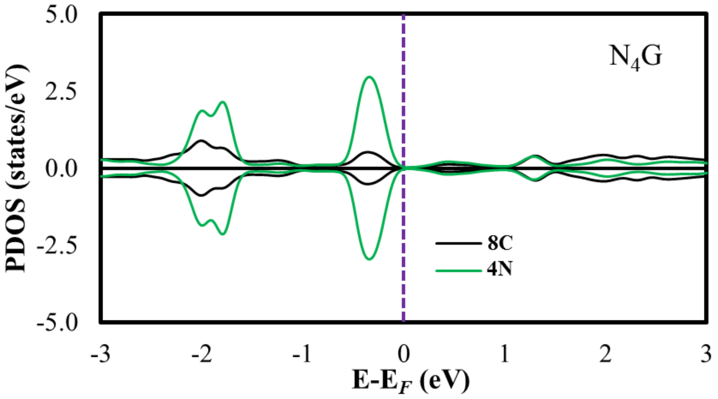

Figure S2. PDOS plots of valence *p*-states of N_4G. Positive and negative values of PDOS indicate the spin-up and spin-down states, respectively.

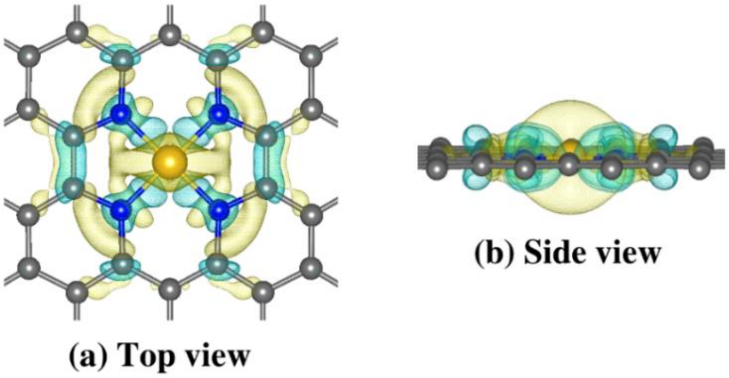

Figure S3. SiN_4G model with charge density difference after and before Si incorporated in N_4G. The light blue and yellow basins represent electron increment and reduction, respectively. The isosurface values of charge differences are $\pm 5x10^{-3}$ |e|/Å^3.

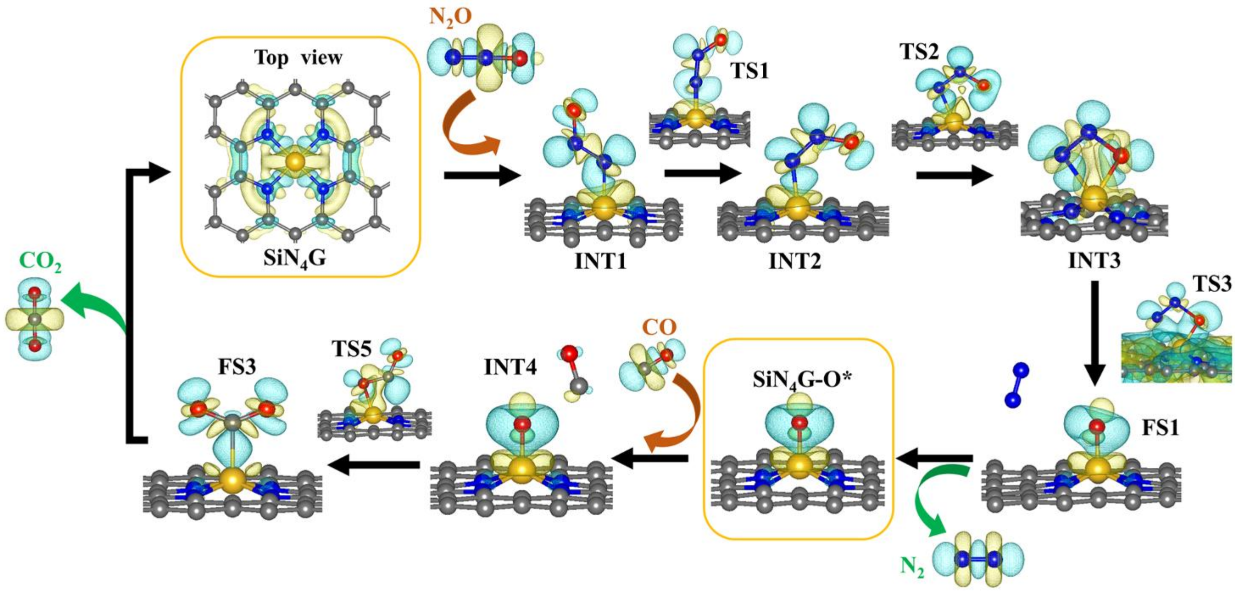

Figure S4. Charge differences of structures in the most favorable pathway (Path 1A and 2B). The light blue and yellow basins represent electron increment and reduction, respectively. The isosurface values of charge differences are $\pm$ $5x10^{-3}$ |e|/Å^3.

Table S1 Bader change (in |e|) of atoms in the system

System	Atom of adsorbent	Charge |e|	Atom of adsorbate	Charge |e|
**SiN_4G**	Si<br>N1<br>N2<br>N3<br>N4	+2.66<br>-1.39<br>-1.52<br>-1.47<br>-1.47		
**SiN_4G-O**	Si<br>N1<br>N2<br>N3<br>N4	+2.95<br>-1.39<br>-1.39<br>-1.38<br>-1.34	O	-1.39
**SiN_4G-N_2O**	Si<br>N1<br>N2<br>N3<br>N4	+2.94<br>-1.46<br>-1.45<br>-1.52<br>-1.50	N1' (bound with Si)<br>N2'<br>O	-0.85<br>+0.18<br>-0.36
**SiN4G-CO_2 (C-bind)**	Si<br>N1<br>N2<br>N3<br>N4	+2.70<br>-1.40<br>-1.37<br>-1.40<br>-1.36	C' (bound with Si)<br>O1'<br>O2'	+1.15<br>-1.11<br>-1.10
**N_2O (gas)**			N1'<br>N2'<br>O	+1.32<br>-0.99<br>-0.34
**N_2 (gas)**			N<br>N	±0.21<br>±0.21
**CO (gas)**			C<br>O	+1.71<br>-1.71
**CO_2 (gas)**			C<br>O1<br>O2	+3.26<br>-1.63<br>-1.63

3. Additional pathway

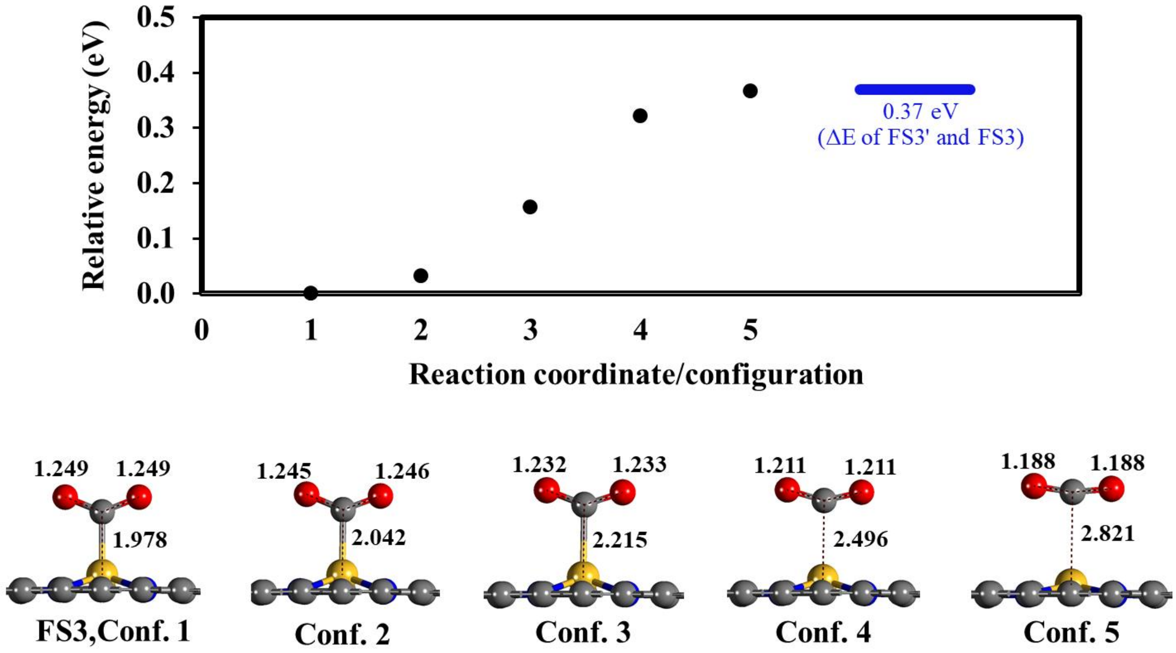

Figure S5. The profile energy of the FS3 to FS3' step and corresponding configurations
